# Real-world safety of terlipressin in elderly patients with liver cirrhosis and esophagogastric variceal bleeding: a focus on hyponatremia and administration methods

**DOI:** 10.3389/fphar.2026.1861406

**Published:** 2026-08-03

**Authors:** Yifei Peng, Jialin Li, Meng Li, Hongxia Duan, Rui Peng, Ping Zuo

**Affiliations:** Leshan Hospital of Traditional Chinese Medicine, Affiliated to Chengdu University of Traditional Chinese Medicine, Leshan, Sichuan, China

**Keywords:** elderly, esophageal and gastric variceal bleeding, hyponatremia, liver cirrhosis, omeprazole, real-world study, terlipressin

## Abstract

**Introduction:**

Terlipressin is a first-line vasoactive agent for esophageal and gastric variceal bleeding (EGVB) in patients with liver cirrhosis, typically administered alongside proton pump inhibitors as background acid-suppressive therapy. Despite its widespread use, there is limited real-world safety data concerning its effects on the elderly population. This study aims to assess the real-world safety of terlipressin in elderly EGVB patients, with omeprazole administered as standard background therapy, specifically examining the incidence and risk factors associated with hyponatremia and acute kidney injury (AKI).

**Methods:**

We conducted a single-center retrospective cohort study involving 90 elderly cirrhotic patients (aged ≥60 years) with EGVB. These patients received a combination of terlipressin and omeprazole at Leshan Hospital of Traditional Chinese Medicine between 1 January 2023, and 31 December 2025. The database was finalized on 31 January 2026, with all patients either discharged or deceased, ensuring no loss to follow-up. We collected data on demographic characteristics, liver function reserve, laboratory indicators, efficacy, and safety indicators. To identify risk factors for hyponatremia, we employed multivariate logistic regression analysis. Additionally, we performed an exploratory comparison of safety differences between two administration methods: intermittent intravenous push and continuous pump infusion. This analysis was *post hoc* and exploratory; given the small sample size and non-random grouping, the findings should be interpreted with caution.

**Results:**

The study included 90 patients with an average age of 68.5 ± 6.2 years. Males comprised 70.0% of the cohort, and 38.9% were classified as Child-Pugh class C. The hemostasis success rate within 72 h was 98.9% (89/90), while the rebleeding rate within 5 days was 13.5% (12/89). The in-hospital mortality rate stood at 10.0% (9/90). Hyponatremia occurred in 68.9% (62/90) of patients, with moderate and severe cases (serum sodium <130 mmol/L) accounting for 37.8% (34/90). The incidence of AKI was 17.8% (16/90). Multivariate logistic regression analysis revealed that Child-Pugh class C (OR = 3.12, 95%CI 1.08–9.02, P = 0.03) and reduced baseline serum sodium (per 5 mmol/L decrease, OR = 2.05, 95%CI 1.21–3.47, P = 0.008) were independent risk factors for hyponatremia. Exploratory subgroup analysis showed that the incidence of moderate-to-severe hyponatremia was numerically lower in the continuous pump infusion group than in the intermittent push group (29.2% vs. 51.5%, nominal P = 0.04, uncorrected for multiple comparisons), but there was no significant difference in overall hyponatremia (P = 0.19). Due to the small sample size, this finding requires prospective validation.

**Conclusion:**

Terlipressin demonstrates a clear hemostatic effect in elderly patients with liver cirrhosis and EGVB when administered with standard background therapy including omeprazole. However, it is associated with a relatively high incidence of hyponatremia. Patients classified as Child-Pugh class C or those with low baseline serum sodium levels require enhanced monitoring of serum sodium. The impact of administration method on hyponatremia requires further investigation.

## 1 Introduction

Esophagogastric variceal bleeding (EGVB) is one of the most critical complications of cirrhotic portal hypertension, characterized by sudden onset, massive bleeding, and high mortality, posing a serious threat to patients’ lives (Kumar et al., 2023). Statistics show that approximately 30% of patients with liver cirrhosis already have esophageal varices at diagnosis, and this proportion can rise to 90% within 10 years, with an annual first bleeding rate of 5%–15% (de Franchis et al., 2022). Although comprehensive treatment methods such as endoscopic therapy, vasoactive drugs, and transjugular intrahepatic portosystemic shunt have continuously improved in recent years, the mortality rate of acute EGVB remains high, and the risk of rebleeding within 6 weeks after the first bleeding is as high as 30%–40% (EASL, 2022; Kaplan et al., 2024). Therefore, how to quickly and effectively control bleeding and prevent rebleeding while minimizing treatment-related adverse drug events is a key issue in clinical practice.

China is a highly endemic area for hepatitis B virus infection, and hepatitis B-related cirrhosis remains the main cause of EGVB (Xiao et al., 2019). As the population ages, the proportion of elderly patients with liver cirrhosis is increasing year by year. Compared with young and middle-aged patients, the elderly often have multiple underlying diseases (hypertension, diabetes, chronic kidney disease, etc.), weakened liver metabolic function, and significantly reduced tolerance to drug metabolism and hemodynamic changes (Elsebaey et al., 2018; Maher et al., 2021). Moreover, elderly individuals often have physiological changes such as decreased vascular elasticity, reduced renal function, and weakened electrolyte regulation ability, making the risk-benefit ratio of drug therapy more complex (Corsonello et al., 2020). Therefore, exploring safer and more individualized treatment strategies in elderly patients with cirrhotic EGVB is of great clinical significance.

Terlipressin is a synthetic vasopressin analog that selectively activates V1 receptors to cause splanchnic vasoconstriction, reduce portal blood flow and pressure, while partially activating V2 receptors to produce antidiuretic effects, thereby effectively controlling acute EGVB (Liu et al., 2025). Multiple randomized controlled trials (RCTs) and systematic reviews have confirmed that terlipressin combined with endoscopic therapy can significantly improve hemostasis success rates and reduce rebleeding rates, with efficacy superior to placebo and comparable to somatostatin or octreotide (Hassan et al., 2023; Rehman et al., 2024). Based on this evidence, domestic and international guidelines have listed terlipressin as a first-line vasoactive drug for acute EGVB, recommending its use as early as possible after suspected or confirmed bleeding (de Franchis et al., 2022; Kaplan et al., 2024).

However, existing RCTs generally have strict inclusion and exclusion criteria: most studies excluded patients aged >70 years, with baseline serum creatinine >2.0 mg/dL, severe cardiovascular disease, hepatic encephalopathy grades 3–4, and those with other organ failures (Jindal et al., 2024). This means that the high-level evidence on which current guidelines are based almost entirely comes from “idealized” trial populations, while the elderly, multimorbid, and organ-dysfunctional patients who most need treatment in real-world clinical practice are excluded. Taking terlipressin as an example, real-world safety and efficacy data in the elderly population remain scarce. In recent years, real-world studies from the UK and the US have shown that terlipressin is often used in combination with albumin for the treatment of hepatorenal syndrome in clinical practice, but data on elderly patients with EGVB remain extremely limited (Velez et al., 2023; Biggins et al., 2025). Furthermore, adverse reactions to terlipressin, particularly hyponatremia and acute kidney injury (AKI), may be more prominent in elderly patients (Thomson et al., 2022; Arora et al., 2023). Moreover, the optimal administration method of terlipressin—intermittent bolus versus continuous infusion—remains an area of ongoing investigation (Şenkaya et al., 2025). Currently, there are no large-sample real-world studies specifically targeting the elderly cirrhotic EGVB population, and clinicians lack reliable safety references when making medication decisions.

Based on the above background, this study aims to evaluate the real-world safety of terlipressin in elderly (≥60 years) patients with cirrhotic EGVB through a single-center retrospective cohort study, with omeprazole administered as standard background therapy. The study focuses on the incidence, severity, and risk factors of hyponatremia and AKI, to provide evidence-based support for individualized clinical medication.

## 2 Methods

### 2.1 Study design and patient selection

This study is a single-center retrospective cohort study that consecutively enrolled elderly patients with cirrhotic EGVB hospitalized at Leshan Hospital of Traditional Chinese Medicine from 1 January 2023, to 31 December 2025. Data were locked on 31 January 2026, and all patients had been discharged or died, with no loss to follow-up. Inclusion criteria: (1) age ≥60 years; (2) clinically diagnosed with liver cirrhosis and confirmed by endoscopy as EGVB; (3) received terlipressin combined with omeprazole for ≥48 h during hospitalization; (4) complete clinical data. In line with the definition commonly used in Chinese national health guidelines, ‘elderly’ was defined as age ≥60 years. This threshold differs from the ≥65 years standard often adopted in Western literature, which should be considered when comparing results across studies. Exclusion criteria: (1) combined with liver cancer or other malignant tumors; (2) combined with hematological diseases; (3) presence of severe renal insufficiency (baseline serum creatinine >2.5 mg/dL) or severe hyponatremia (serum sodium <125 mmol/L) at admission.

This study is a retrospective observational study, and all data were anonymized and extracted before ethical review, without any clinical intervention on patients. The Medical Ethics Committee of Leshan Hospital of Traditional Chinese Medicine approved the use of the collected data for analysis on 19 March 2026 (in accordance with Chinese regulations on retrospective research ethics review), and waived the requirement for patient informed consent (Ethics Approval No.: 202602). The committee confirmed that the study complies with the principles of the Declaration of Helsinki regarding retrospective data research.

**FIGURE 1:**
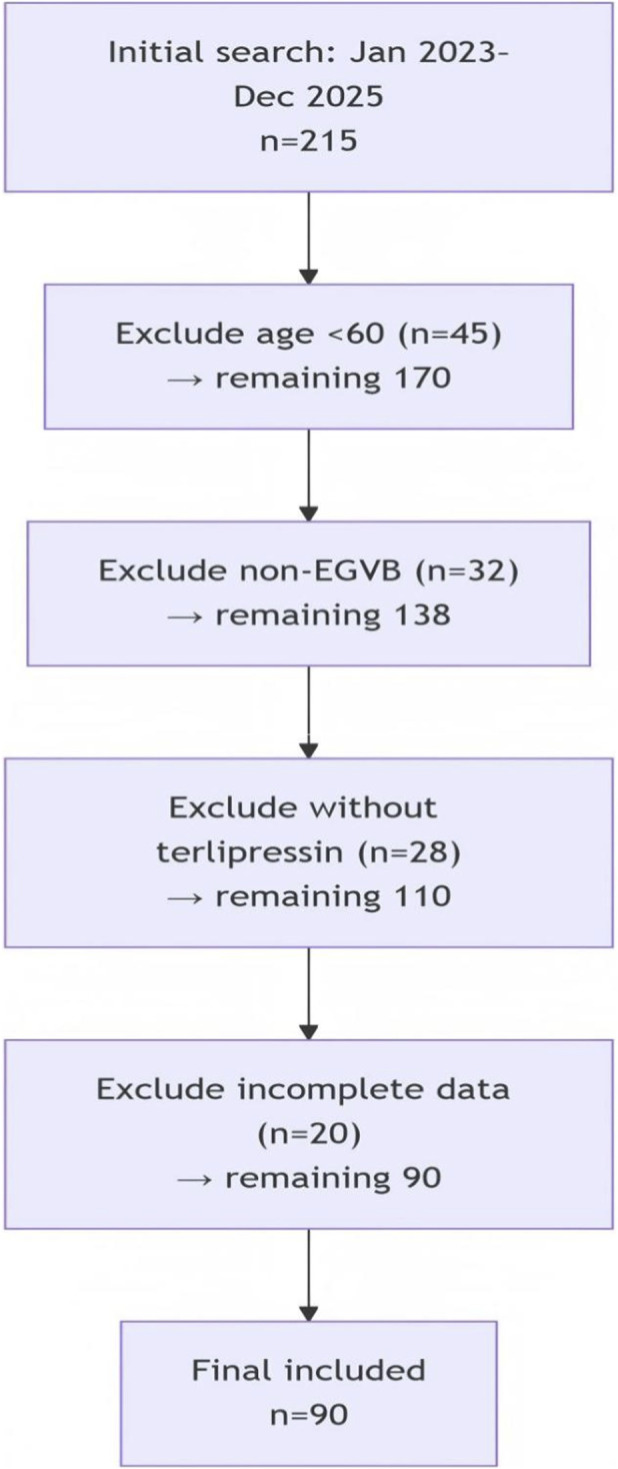
Patient screening flowchart (Flowchart: Initial retrieval of cirrhotic bleeding inpatient records (n = 215) → Exclude age <60 years (n = 45) → Exclude non-EGVB bleeding (n = 32) → Exclude those not using terlipressin (n = 28) → Exclude incomplete data (n = 20) → Final inclusion for analysis (n = 90).

Patient screening process: Initial retrieval of 215 inpatient medical records of cirrhotic bleeding from 1 January 2023, to 31 December 2025, excluded 45 cases aged <60 years, 32 cases of non-EGVB bleeding, 28 cases not using terlipressin, and 20 cases with incomplete data, finally including 90 cases (Figure 1). Among key variables, serum sodium was missing in 3 cases (3.3%), and serum creatinine was missing in 2 cases (2.2%), with no overlapping missing cases; these were directly excluded, resulting in a final effective sample size of 90. The excluded five patients showed no significant differences from the included patients in age (68.1 ± 7.0 vs. 68.5 ± 6.2, P = 0.82), gender (male 60% vs. 70%, P = 0.64), or Child-Pugh classification (class C 40% vs. 38.9%, P = 0.96), suggesting low exclusion bias.

### 2.2 Treatment regimen

All patients received standard basic treatment for cirrhotic EGVB, including fasting, oxygen therapy, volume expansion, blood transfusion (packed red blood cells, according to hemoglobin levels and hemodynamic status), prophylactic antibiotics (third-generation cephalosporins or fluoroquinolones for 3–5 days), and close monitoring of vital signs.

The use of terlipressin followed the following strategy:

Timing of initiation: Immediately after diagnosis of EGVB, no later than 6 h after admission.

Administration method: Divided into two regimens based on clinician decision.

Intermittent intravenous push group (66 cases, 73.3%): Initial intravenous push of 250 μg, followed by 1–2 mg intravenously every 4–6 h, with specific intervals and doses determined by the attending physician based on the patient’s blood pressure, heart rate, and bleeding activity.

Continuous pump infusion group (24 cases, 26.7%): After an initial intravenous push of 250 μg, continuous intravenous pump infusion at a rate of 250 μg/h, with infusion rate adjustment based on hemodynamic response during maintenance treatment, maximum infusion rate not exceeding 500 μg/h.

Course of treatment: Continuous use for 3–5 days. If no rebleeding occurred within 72 h and hemodynamics were stable, treatment could be discontinued early; if active bleeding or risk of rebleeding persisted after 5 days, the attending physician could evaluate whether to extend the course, up to a maximum of 7 days.

Dose adjustment and discontinuation criteria: If the following occurred during treatment, dose reduction (e.g., extending intermittent push to 1 mg every 6 h, reducing continuous pump to 125 μg/h) or discontinuation was considered:

Significant increase in blood pressure (systolic blood pressure >160 mmHg) or hypertensive crisis; New-onset arrhythmia (especially bradycardia <50 beats/min); Decrease in serum sodium >10 mmol/L or serum sodium <125 mmol/L; Manifestations of severe vasospasm such as myocardial ischemia and intestinal ischemia.

Indications for drug withdrawal: Completion of the prescribed treatment course, occurrence of serious adverse events, patient death, or transfer to another hospital.

Intravenous omeprazole (40 mg, 1–2 times daily for 3–5 days) was administered as standard background acid-suppressive therapy for acute variceal bleeding, in accordance with current guidelines. The choice, dose, and duration were at the discretion of treating physicians and were not part of the study’s comparative analysis. In this study, the focus was solely on the intravenous administration of omeprazole during hospitalization. Oral medication taken after discharge was not included in the evaluation. Although some patients continued oral omeprazole post-discharge, our analysis concentrated on the medication regimen during hospitalization. The “number of days of omeprazole use” in the table reflects only the days of intravenous administration during hospitalization, with a mean duration of 3.8 ± 1.5 days.

### 2.3 Data collection

Data were collected from the electronic medical record system and included the following: (1) demographic characteristics; (2) etiology of liver cirrhosis; (3) liver function reserve, assessed by Child-Pugh classification and MELD score; (4) comorbidities; (5) baseline laboratory indicators such as hemoglobin, platelets, serum sodium, serum creatinine, eGFR, urea, ALT, AST, total bilirubin, albumin, prothrombin time, and INR; (6) severity of bleeding; (7) medication status; (8) efficacy indicators, including successful hemostasis within 24 h and 72 h, rebleeding within 5 days and during hospitalization, length of hospital stay, and in-hospital death; (9) safety indicators, which encompassed the lowest serum sodium value during treatment, decrease in serum sodium, hyponatremia classification, peak serum creatinine, AKI stage (according to KDIGO criteria), new-onset arrhythmia, sinus bradycardia, and myocardial ischemia.

### 2.4 Data quality control

Two researchers independently extracted and cross-checked all data. Any discrepancies were resolved through discussion by consulting the original medical records. Missing data were not supplemented; instead, only the effective sample size was reported. Outliers were identified using box plots, and the original records were subsequently rechecked.

### 2.5 Outcome indicators

The primary efficacy endpoint was the hemostasis success rate within 72 h, while the primary safety endpoints included incidences of hyponatremia and acute kidney injury (AKI). Hemostasis success within this timeframe was defined by meeting all of the following criteria after the initial administration of terlipressin: (1) Hemodynamic stability, indicated by a systolic blood pressure of at least 90 mmHg and a heart rate of 100 beats per minute or less, without the need for vasoactive drugs; (2) Absence of clinical signs of active bleeding, such as hematemesis, melena, or clear gastric tube drainage; (3) Stable hemoglobin levels or a decrease of less than 20 g/L; (4) No requirement for additional hemostatic interventions, including endoscopy, interventional therapy, or surgery.

Rebleeding within 5 days was defined by the occurrence of any of the following conditions after achieving successful hemostasis within 72 h: (1) New-onset hematemesis or melena; (2) A hemoglobin decrease of 20 g/L or more necessitating blood transfusion; (3) The need for repeated endoscopic or interventional hemostasis.

Hyponatremia, characterized by a serum sodium level below 135 mmol/L, is categorized into mild (130–134 mmol/L), moderate (125–129 mmol/L), and severe (<125 mmol/L) based on the lowest recorded value. Acute Kidney Injury (AKI) was defined and staged following the KDIGO serum creatinine criteria. Due to the retrospective design and incomplete hourly urine output records for a substantial proportion of patients, the urine output criterion could not be reliably applied. Therefore, AKI diagnosis and staging in this study were based solely on serum creatinine changes.

Shock is characterized by a systolic blood pressure of less than 90 mmHg or a reduction of 40 mmHg or more from baseline, along with signs of inadequate tissue perfusion, such as altered consciousness and urine output below 0.5 mL/kg/h.

Endoscopic active bleeding is classified as Forrest grade Ia (spurting bleeding) or Ib (oozing bleeding).

The formula for calculating the MELD score is: MELD = 9.57 × ln (creatinine in mg/dL) + 3.78 × ln (bilirubin in mg/dL) + 11.2 × ln (INR) + 6.43. When creatinine and bilirubin levels are below 1.0 mg/dL, they should be adjusted to 1.0 for the calculation.

### 2.6 Statistical methods

Statistical analysis employed SPSS 26.0 software. Continuous variables following a normal distribution were represented as mean ± standard deviation, and comparisons between groups utilized the independent-sample t-test. For paired comparisons of baseline and nadir laboratory values within the same patient, a paired-samples t-test was used. For continuous variables not following a normal distribution, data were expressed as median (interquartile range), with the Mann-Whitney U test used for group comparisons.

The expected incidence of hyponatremia, the primary endpoint, was approximately 50% based on previous literature. With α set at 0.05 and an allowable error of 10%, the minimum sample size was estimated at 96 cases using the sample size calculation formula for a single-group rate. In this study, 90 cases were included, which fell short of the pre-specified target.

A *post hoc* precision analysis was conducted for the primary endpoint of hyponatremia incidence. With 90 patients and an observed hyponatremia rate of 68.9%, the achieved 95% confidence interval width, calculated using the Clopper-Pearson exact method, was ±9.9 percentage points (58.3%–78.2%). This indicates that the study retains adequate precision for the main incidence estimate. However, the shortfall from the target of 96 cases compromises statistical power for secondary and subgroup analyses.

For acute kidney injury (AKI), with only 16 cases observed, only descriptive statistics were performed without inter-group comparisons or trend inferences.

A multivariate logistic regression analysis identified risk factors for hyponatremia. Based on prior literature and clinical expertise, the pre-specified variables included Child-Pugh classification, baseline serum sodium, MELD score, and shock on admission. Variables with a P-value of less than 0.10 in the univariate analysis, specifically Child-Pugh class C and baseline serum sodium, were incorporated into the multivariate model. Odds ratios (OR) and their 95% confidence intervals (CI) were calculated. The model’s goodness-of-fit was evaluated using the Hosmer-Lemeshow test, while the area under the receiver operating characteristic curve (AUC) and its 95%CI were used to assess the model’s apparent discriminative ability. The reported AUC was derived from the same dataset used to build the model and was not corrected for optimism (e.g., via bootstrap or cross-validation). Hemostasis time curves were plotted using the Kaplan-Meier method, and group differences were compared with the log-rank test. All tests were two-sided, with a P-value of less than 0.05 considered statistically significant.

The subgroup analysis of administration methods, comparing intermittent intravenous push with continuous pump infusion, was a *post hoc* exploratory study. Given the small sample size of only 24 cases in the continuous pump infusion group and the non-randomized grouping, these results should be interpreted cautiously and considered as preliminary data indications. To control for confounding factors, propensity score matching (PSM) was employed for sensitivity analysis. The matching variables included age, gender, Child-Pugh classification, MELD score, and baseline serum sodium. The nearest-neighbor matching method, with a caliper value of 0.2 times the standard deviation, was used for 1:1 matching. Given that multiple safety endpoints were compared in this *post hoc* subgroup analysis, no multiplicity adjustment was applied. All reported P-values for this analysis are therefore nominal and must be interpreted with caution, as they carry a high risk of Type I error.

## 3 Results

### 3.1 Baseline characteristics

The study included 90 patients, with an average age of 68.5 ± 6.2 years, and 70.0% were male. Hepatitis B was the primary cause of liver cirrhosis, affecting 60.0% of the participants. According to the Child-Pugh classification, 50.0% of the patients were in class B, while 38.9% were in class C. The average MELD score was 15.2 ± 7.8. Regarding comorbidities, 20.0% of the patients had diabetes, and 24.4% had chronic kidney disease (eGFR <60). Shock on admission was present in 53.3% (48/90) of patients. Baseline laboratory indicators and bleeding severity are presented in Table 1.

**TABLE 1:** Baseline characteristics of enrolled patients (N = 90).

Variable	Value
Demographic characteristics	
Age (years), mean ± SD	68.5 ± 6.2
Age ≥75 years, n (%)	18 (20.0)
Male, n (%)	63 (70.0)
Etiology of cirrhosis, n (%)	​
Hepatitis B	54 (60.0)
Hepatitis C	5 (5.6)
Alcoholic	20 (22.2)
Autoimmune	9 (10.0)
Other/cryptogenic	2 (2.2)
Liver function reserve	
Child-pugh classification, n (%)	
Class A	10 (11.1)
Class B	45 (50.0)
Class C	35 (38.9)
MELD score, mean ± SD	15.2 ± 7.8
MELD ≥18, n (%)	28 (31.1)
Comorbidities, n (%)	
Hypertension	14 (15.6)
Diabetes	18 (20.0)
Coronary artery disease	7 (7.8)
Heart failure	5 (5.6)
Atrial fibrillation	6 (6.7)
Chronic kidney disease (eGFR<60)	22 (24.4)
Baseline laboratory indicators, mean ± SD	
Hemoglobin (g/L)	86.2 ± 21.5
Platelets (×10^9^/L)	88.3 ± 55.2
Serum sodium (mmol/L)	137.2 ± 4.3
Serum creatinine (μmol/L)	92.5 ± 58.2
eGFR (mL/min/1.73 m^2^)	74.3 ± 18.7
Urea (mmol/L)	10.1 ± 5.6
ALT (U/L)	62.4 ± 48.1
AST (U/L)	55.3 ± 36.2
Total bilirubin (μmol/L)	40.5 ± 34.2
Albumin (g/L)	30.1 ± 5.3
PT (s)	15.2 ± 3.1
INR	1.35 ± 0.32
Severity of bleeding	
Shock on admission, n (%)	48 (53.3)
Lowest hemoglobin (g/L), mean ± SD	66.5 ± 14.8
Red blood cell transfusion, n (%)	62 (68.9)
Transfusion volume (U), mean ± SD	4.2 ± 2.5
Active bleeding on endoscopy, n (%)	68 (75.6)
Medication status	
Days of terlipressin use, mean ± SD	3.4 ± 1.7
Daily dose (mg/day), mean ± SD	2.6 ± 0.8
Daily dose in intermittent push group (mg/day)	2.5 ± 0.7
Daily dose in continuous infusion group (mg/day)	2.9 ± 0.9
Comparison of daily doses between groups	P = 0.12
Administration method, n (%)	
Intermittent push	66 (73.3)
Continuous pump infusion	24 (26.7)
Days of omeprazole use (intravenous during hospitalization), mean ± SD	3.8 ± 1.5

Comparison of daily doses between the two groups was performed using the independent-samples t-test.

### 3.2 Efficacy indicators

At 72 h, the hemostasis success rate was 98.9% (89/90), while at 24 h, it was 84.4% (76/90). The rebleeding rate within 5 days stood at 13.5% (12/89), and during hospitalization, it was 17.8% (16/90). Patients had an average hospital stay of 13.5 ± 6.2 days, with an in-hospital mortality rate of 10.0% (9/90). Of the nine in-hospital deaths, none was directly attributed to terlipressin-related adverse effects, including severe hyponatremia, acute kidney injury, or cardiovascular events. The deaths were primarily due to uncontrolled variceal bleeding, decompensated liver failure, and sepsis-related multi-organ failure. The median time to hemostasis was 8.5 h, and the cumulative hemostasis rate at 72 h was 98.9% (Table 2).

**TABLE 2:** Efficacy indicators (N = 90).

Indicator	Value
24 h hemostasis success, n (%)	76 (84.4)
72 h hemostasis success, n (%)	89 (98.9)
5-day rebleeding, n (%)	12 (13.5)
In-hospital rebleeding, n (%)	16 (17.8)
Hospital stay (days), mean ± SD	13.5 ± 6.2
In-hospital death, n (%)	9 (10.0)

The denominator for 5-day rebleeding was the 89 patients who achieved hemostasis within 72 h.

### 3.3 Safety indicators

**FIGURE 2:**
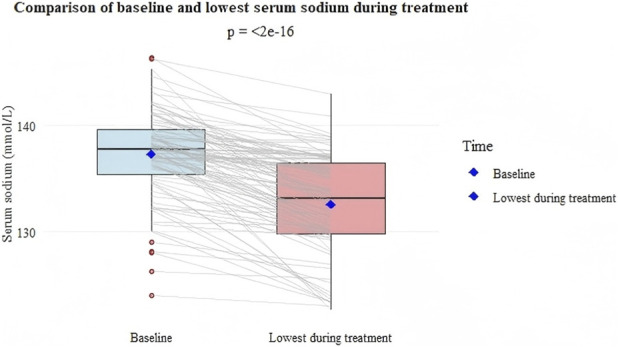
Box plot of baseline and lowest serum sodium during treatment (Paired comparison of baseline serum sodium and the lowest serum sodium during treatment in 90 patients. Baseline serum sodium: 137.2 ± 4.3 mmol/L, lowest serum sodium during treatment: 131.2 ± 4.5 mmol/L, paired t-test P < 0.001).

During the treatment period, the average minimum serum sodium level was 131.2 ± 4.5 mmol/L (Figure 2), with an average reduction of 6.0 ± 4.2 mmol/L. The incidence of hyponatremia, defined as serum sodium levels below 135 mmol/L, was 68.9% (62 out of 90 patients). Among these cases, mild hyponatremia occurred in 31.1% (28 out of 90), moderate hyponatremia in 26.7% (24 out of 90), and severe hyponatremia in 11.1% (10 out of 90). Additionally, 60.0% (54 out of 90) of patients experienced a serum sodium decrease of 5 mmol/L or more, while 26.7% (24 out of 90) had a decrease of 10 mmol/L or more (Table 3).

**TABLE 3:** Safety indicators (N = 90).

Indicator	Value
Hyponatremia	
Lowest serum sodium during treatment (mmol/L), mean ± SD	131.2 ± 4.5
Decrease in serum sodium (mmol/L), mean ± SD	6.0 ± 4.2
Any hyponatremia (<135 mmol/L), n (%)	62 (68.9)
Mild (130–134 mmol/L)	28 (31.1)
Moderate (125–129 mmol/L)	24 (26.7)
Severe (<125 mmol/L)	10 (11.1)
Decrease in serum sodium ≥5 mmol/L, n (%)	54 (60.0)
Decrease in serum sodium ≥10 mmol/L, n (%)	24 (26.7)
Acute kidney injury (AKI)	
Peak serum creatinine (μmol/L), mean ± SD	115.3 ± 98.7
Any AKI, n (%)	16 (17.8)
AKI stage 1	12 (13.3)
AKI stage 2	3 (3.3)
AKI stage 3	1 (1.1)
RRT required, n (%)	0
Cardiac events	
New-onset arrhythmia, n (%)	0
Sinus bradycardia, n (%)	0
Myocardial ischemia, n (%)	0
Other serious adverse events	
Lactic acidosis, n (%)	0
Intestinal ischemia, n (%)	0
Severe vasospasm, n (%)	0
Drug discontinuation due to adverse events, n (%)	1 (1.1)

The incidence of AKI was 17.8% (16/90), with stage 1 comprising 13.3% (12/90), stage 2 at 3.3% (3/90), and stage 3 at 1.1% (1/90). AKI staging was based solely on serum creatinine changes per KDIGO criteria, without the urine output criterion. None of the cases required RRT. Given the limited number of AKI events, this report remains descriptive without inter-group comparisons or trend inferences. Regarding cardiac events, there were no instances of new-onset arrhythmia, sinus bradycardia, or myocardial ischemia. One case (1.1%) discontinued the drug due to adverse events.

### 3.4 Subgroup analysis: efficacy and safety by Child-Pugh classification

The incidence of any hyponatremia was significantly higher in patients with Child-Pugh class C compared to those with class A/B, with rates of 82.9% versus 60.0% (P = 0.02). Similarly, the incidence of moderate or severe hyponatremia was 54.3% in class C patients, compared to 27.3% in class A/B patients (P = 0.01). The incidences of acute kidney injury (AKI) were 25.7% (9 out of 35) in the class C group and 12.7% (7 out of 55) in the class A/B group. Due to the limited number of events, only the values are presented here without statistical inference (Table 4).

**TABLE 4:** Comparison of efficacy and safety by Child-Pugh classification.

Indicator	Child-pugh A/B (n = 55)	Child-pugh C (n = 35)	P value
72 h hemostasis success, n (%)	55 (100)	34 (97.1)	0.27
5-day rebleeding, n (%)	5 (9.1)	7 (20.6)	0.12
Any hyponatremia, n (%)	33 (60.0)	29 (82.9)	0.02
Moderate or severe hyponatremia, n (%)	15 (27.3)	19 (54.3)	0.01
Any AKI, n (%)	7 (12.7)	9 (25.7)	Descriptive

### 3.5 Exploratory analysis: safety comparison by administration method

To further investigate the optimal dosing strategy for elderly patients, we performed a *post hoc* exploratory subgroup analysis comparing baseline characteristics and safety indicators between the intermittent intravenous push group (n = 66) and the continuous pump infusion group (n = 24). Given the non-random assignment and significant sample size disparity between the groups, these findings are preliminary and should be interpreted cautiously. No significant differences were observed between the groups in terms of age, gender, Child-Pugh classification, MELD score, and baseline serum sodium (Table 5). Safety comparisons revealed that the incidence of hyponatremia was 72.7% (48/66) in the intermittent group and 58.3% (14/24) in the continuous group, with no statistically significant difference (P = 0.19). However, the incidence of moderate to severe hyponatremia (serum sodium <130 mmol/L) was numerically lower in the continuous pump infusion group compared to the intermittent group (29.2% vs. 51.5%, nominal P = 0.04, uncorrected for multiple comparisons and therefore at high risk of Type I error). The incidence of acute kidney injury (AKI) was 18.2% in the intermittent group and 16.7% in the continuous group. Due to the limited number of events, further interpretation was not possible. Detailed safety outcomes by administration method are summarized in Table 6.

**TABLE 5:** Comparison of baseline characteristics by administration method.

Variable	Intermittent push group (n = 66)	Continuous infusion group (n = 24)	P value
Age (years), mean ± SD	68.1 ± 6.5	69.5 ± 5.3	0.32
Male, n (%)	46 (69.7)	17 (70.8)	0.92
Child-pugh class C, n (%)	25 (37.9)	10 (41.7)	0.74
MELD score, mean ± SD	15.0 ± 7.5	15.8 ± 8.2	0.66
Baseline serum sodium (mmol/L), mean ± SD	137.0 ± 4.5	137.8 ± 3.8	0.44
Baseline serum creatinine (μmol/L), mean ± SD	91.2 ± 57.6	96.3 ± 60.2	0.71

**TABLE 6:** Comparison of safety outcomes by administration method.

Safety outcome	Intermittent push group (n = 66)	Continuous infusion group (n = 24)	P value
Any hyponatremia (serum sodium <135 mmol/L), n (%)	48 (72.7)	14 (58.3)	0.19
Moderate-to-severe hyponatremia (serum sodium <130 mmol/L), n (%)	34 (51.5)	7 (29.2)	0.04*
Serum sodium decrease (mmol/L), mean ± SD	6.2 ± 4.3	5.4 ± 3.9	0.42
Any AKI, n (%)	12 (18.2)	4 (16.7)	0.87
Drug discontinuation due to adverse events, n (%)	1 (1.5)	0 (0.0)	1.00

* Nominal P value; uncorrected for multiple comparisons.

### 3.6 Sensitivity analysis: propensity score matching (PSM)

To address baseline differences between the two groups, we employed propensity score matching (PSM) for sensitivity analysis. The matching variables included age, gender, Child-Pugh classification, MELD score, and baseline serum sodium. We utilized the nearest-neighbor matching method with a caliper value set at 0.2 times the standard deviation. Following 1:1 matching, each group—the continuous infusion group and the intermittent intravenous bolus group—comprised 18 cases. Table 7 presents the comparison of baseline characteristics between the two groups post-matching. After PSM, the incidence of moderate-severe hyponatremia was 28.6% in the continuous infusion group and 50.0% in the intermittent bolus group (nominal P = 0.04). This result is derived from a subset of the same data with only 18 matched pairs, is severely underpowered, and does not constitute independent validation. It should be regarded as exploratory only.

**TABLE 7:** Comparison of baseline characteristics after PSM matching.

Variable	Intermittent push group (n = 18)	Continuous infusion group (n = 18)	P value
Age (years), mean ± SD	68.5 ± 6.1	68.9 ± 5.8	0.83
Male, n (%)	13 (72.2)	12 (66.7)	0.72
Child-Pugh class C, n (%)	7 (38.9)	8 (44.4)	0.74
MELD score, mean ± SD	15.2 ± 7.3	15.5 ± 7.9	0.91
Baseline serum sodium (mmol/L), mean ± SD	137.1 ± 4.4	137.5 ± 3.9	0.77
Baseline serum creatinine (μmol/L), mean ± SD	93.5 ± 56.8	94.2 ± 59.1	0.96

### 3.7 Analysis of risk factors for hyponatremia

**FIGURE 3:**
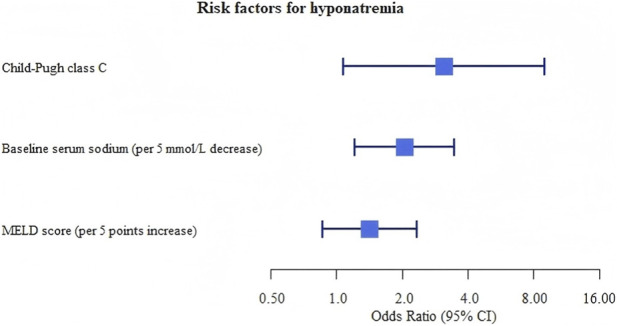
Forest plot of risk factors for hyponatremia (Based on the multivariate logistic regression results in Table 8. The forest plot displays all three variables entered into the final model: Child-Pugh class C (OR = 3.12, 95%CI 1.08–9.02), baseline serum sodium per 5 mmol/L decrease (OR = 2.05, 95%CI 1.21–3.47), and MELD score per 5-point increase (OR = 1.42, 95%CI 0.86–2.34). X-axis is OR (log scale), vertical line at 1).

**FIGURE 4:**
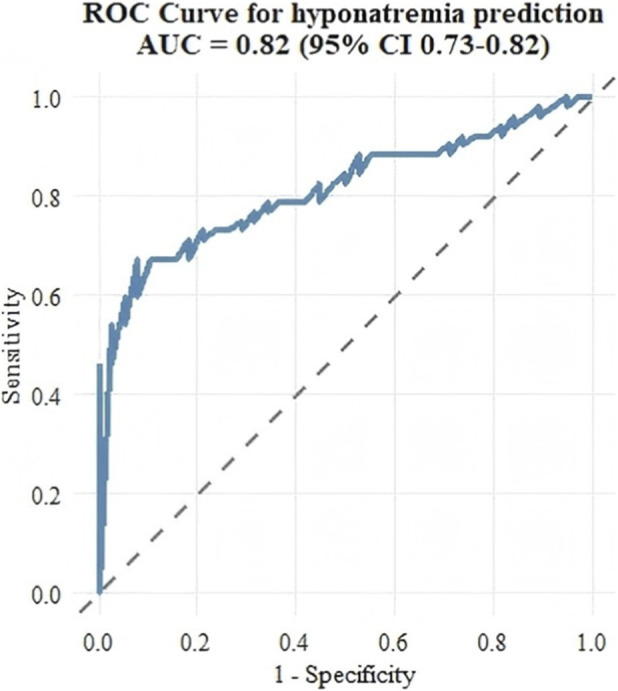
ROC curve of the hyponatremia risk prediction model (Based on the multivariate logistic regression model in Table 8, the ROC curve was plotted with predicted probabilities. The area under the curve (AUC) was 0.76 (95%CI 0.65–0.87), representing apparent discriminative performance derived from the same dataset used to build the model. This AUC has not been corrected for optimism and may overestimate performance in external data. The calibration curve (Figure 6) was generated using bootstrap resampling).

**FIGURE 5:**
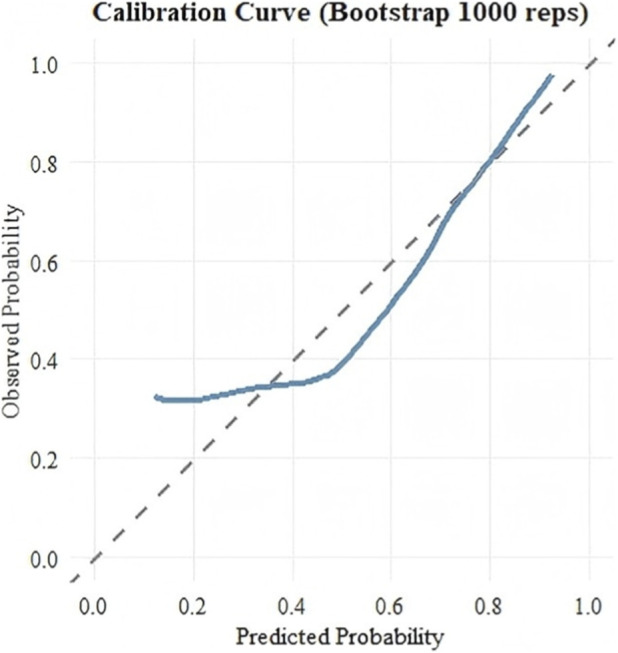
Calibration curve of the hyponatremia risk prediction model (Based on the multivariate logistic regression model in Table 8, the calibration curve was plotted using the bootstrap method (1,000 resamples). The curve shows that the predicted probabilities are generally consistent with the observed probabilities, indicating good calibration).

**FIGURE 6:**
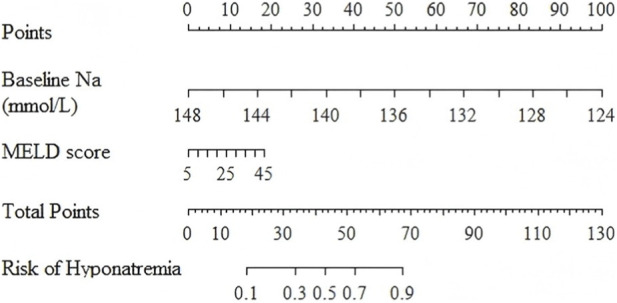
Nomogram for hyponatremia risk (Based on the multivariate logistic regression results in Table 8 a nomogram for predicting hyponatremia risk was constructed. The nomogram includes two predictors: Child-Pugh classification (class C vs. A/B) and baseline serum sodium (mmol/L). The total score is obtained by summing the scores of each variable, corresponding to the predicted probability of hyponatremia. This nomogram has not undergone external validation and requires further research before clinical application).

Univariate analysis indicated that Child-Pugh class C, reduced baseline serum sodium, and elevated MELD score were linked to hyponatremia (P < 0.10). These three variables were incorporated into a multivariate logistic regression model, employing a pre-specified variable selection method grounded in prior literature and clinical expertise. The analysis revealed that Child-Pugh class C (OR = 3.12, 95%CI 1.08–9.02, P = 0.03) and decreased baseline serum sodium (OR = 2.05 for every 5 mmol/L decrease, 95%CI 1.21–3.47, P = 0.008) emerged as independent risk factors for hyponatremia (Figure 3). In contrast, the MELD score did not achieve statistical significance in the multivariate analysis (P = 0.17). The model demonstrated good goodness-of-fit (Hosmer-Lemeshow test P = 0.52) and acceptable discrimination (AUC = 0.76, 95%CI 0.65–0.87). Based on this multivariate logistic regression model, the ROC curve (Figure 4), calibration curve (Figure 5), and nomogram (Figure 6) were generated. The model exhibited good calibration, and the nomogram provided an intuitive assessment of hyponatremia risk in patients. However, this nomogram has not undergone external validation, necessitating further research before clinical application (Table 8).

**TABLE 8:** Multivariate logistic regression analysis of risk factors for hyponatremia.

Variable	Univariate OR (95%CI)	P Value	Multivariate OR (95%CI)	P value
Age (per 10-year increase)	1.32 (0.78–2.23)	0.3	—	—
Female (vs. male)	1.15 (0.45–2.94)	0.77	—	—
Child-Pugh class C	3.45 (1.27–9.36)	0.02	3.12 (1.08–9.02)	0.03
Baseline serum sodium (per 5 mmol/L decrease)	2.18 (1.35–3.52)	0.002	2.05 (1.21–3.47)	0.008
MELD score (per 5-point increase)	1.56 (0.98–2.48)	0.06	1.42 (0.86–2.34)	0.17
Shock on admission	1.23 (0.52–2.91)	0.64	—	—
Continuous pump infusion	1.48 (0.53–4.13)	0.45	—	—
Diabetes	1.96 (0.68–5.65)	0.21	—	—

The multivariable model incorporated variables pre-specified from previous literature and clinical expertise. Ultimately, Child-Pugh class C, baseline serum sodium, and MELD score were included in the model. The Hosmer-Lemeshow test indicated a P-value of 0.52, and the area under the curve (AUC) was 0.76, with a 95% confidence interval of 0.65–0.87.

## 4 Discussion

This study examined real-world data from 90 elderly patients with cirrhotic esophagogastric variceal bleeding (EGVB) treated with terlipressin and standard background therapy. The findings revealed a satisfactory hemostatic effect; however, the incidence of hyponatremia was notably high at 68.9%, and acute kidney injury (AKI) occurred in 17.8% of patients. Multivariate analysis identified Child-Pugh class C and reduced baseline serum sodium as independent risk factors for hyponatremia. An exploratory analysis suggested that continuous pump infusion might offer a numerical advantage over intermittent intravenous bolus injection in mitigating moderate to severe hyponatremia. Nonetheless, this conclusion requires validation through a prospective large-sample study.

### 4.1 Efficacy analysis

In this study, the hemostasis success rate at 72 h was 98.9%, aligning with the 90%–95% success rates reported in previous RCTs (Hassan et al., 2023; Rehman et al., 2024). This suggests that terlipressin is also effective for hemostasis in the elderly population. The rebleeding rate within 5 days was 13.5%, and the in-hospital mortality rate was 10.0%, consistent with the 7%–20% range reported in both domestic and international studies (Zanetto et al., 2021; Garcia-Tsao et al., 2024; Kaplan et al., 2024; Rehman et al., 2024). These findings indicate that terlipressin, administered with standard background therapy, effectively achieves hemostasis in elderly patients with EGVB due to liver cirrhosis.

### 4.2 Safety analysis

The 68.9% incidence of hyponatremia observed in our cohort is notably high, yet direct numerical comparison with figures from landmark randomized controlled trials (RCTs) is methodologically problematic for several reasons. First, pivotal RCTs of terlipressin for EGVB typically excluded elderly patients and those with significant renal or hepatic impairment, enrolling a substantially younger and less severely ill population than ours. Second, the CONFIRM trial (Wong et al., 2021), often cited in terlipressin safety discussions, specifically enrolled patients with hepatorenal syndrome, a distinct clinical entity with different dosing regimens, treatment duration, and baseline fluid/electrolyte status. Third, definitions, monitoring frequency, and reporting thresholds for hyponatremia vary considerably across studies, precluding valid head-to-head comparisons. For these reasons, the 5%–10% hyponatremia rates reported in some RCTs are not appropriate benchmarks for our real-world elderly EGVB cohort. Nonetheless, the substantially higher incidence observed in our elderly cohort, compared with the 5%–10% range typically seen in younger trial populations, underscores the increased susceptibility of older patients to this adverse effect.

A more meaningful comparison can be made with studies focusing on elderly or real-world EGVB populations. Previous studies have reported elevated rates of hyponatremia following terlipressin treatment in cirrhotic patients with variceal bleeding (Han et al., 2019). Our higher rate of 68.9% likely reflects the particularly severe profile of our cohort: 38.9% were classified as Child-Pugh class C, and 53.3% presented with shock on admission, indicating advanced liver disease and hemodynamic instability. Additional contributing factors include: (1) terlipressin-induced V2 receptor activation leading to antidiuresis and dilutional hyponatremia (Liu et al., 2025); (2) age-related decline in renal concentrating ability and water handling capacity (Glassock and Rule, 2022); and (3) the potential influence of fluid management practices, as the types and volumes of fluid resuscitation were not systematically recorded and some patients may have received hypotonic fluids. In this study, moderate to severe hyponatremia accounted for 37.8%, while severe hyponatremia was 11.1%, underscoring the need for heightened clinical vigilance in this vulnerable population.

The incidence of AKI was 17.8%, aligning with the previously reported range of 15%–20% (Belcher et al., 2022). Given the small number of AKI events (16 cases), the study offered only a descriptive report without conducting a multivariate analysis. The findings were presented numerically. Patients with Child-Pugh class C exhibited a higher AKI incidence at 25.7% compared to 12.7% in others. However, due to the limited sample size, the possibility of random fluctuations cannot be ruled out.

### 4.3 Analysis of risk factors

Multivariate analysis revealed that Child-Pugh class C and decreased baseline serum sodium independently predicted hyponatremia risk. This is consistent with previous findings that baseline clinical characteristics are important predictors of hyponatremia risk in patients receiving terlipressin (Han et al., 2019). Patients classified as Child-Pugh class C exhibit severely impaired liver function and diminished drug metabolism, leading to elevated blood drug concentrations and heightened hyponatremia risk. Additionally, low baseline serum sodium indicates existing water-sodium regulation disorders, making further declines more probable post-medication. Monitoring these high-risk groups is crucial.

### 4.4 Exploratory analysis of administration strategies

A *post hoc* exploratory subgroup analysis was conducted to compare intermittent bolus and continuous infusion administration. No significant difference in hemostatic efficacy was observed. A nominally significant difference in moderate-to-severe hyponatremia favoring continuous infusion was noted (nominal P = 0.04, uncorrected for multiple comparisons), and a similar numerical pattern was seen in the 18 matched pairs after PSM. However, given the non-randomized design, the small sample size (only 24 patients in the continuous infusion arm), the absence of multiplicity adjustment, and the severely underpowered post-PSM comparison, these findings cannot support any conclusions about the relative safety of the two administration methods. They serve solely to generate hypotheses for future prospective randomized trials designed specifically to compare intermittent bolus versus continuous infusion of terlipressin in elderly patients with EGVB. Clinicians should base their choice of administration method on individual patient factors and institutional protocols rather than on the exploratory findings reported here.

### 4.5 Clinical recommendations based on study results

Considering the exploratory scope of this study and the limited sample size, definitive clinical recommendations are not currently provided. Nonetheless, drawing from existing literature, it is advised that clinicians exercise caution when administering terlipressin to elderly patients with EGVB:

For patients classified under Child-Pugh class C with low baseline serum sodium, it is crucial to enhance serum sodium monitoring to at least once daily.

If the serum sodium level decreases by more than 10 mmol/L or falls below 130 mmol/L, it is advisable to consider reducing the dose or discontinuing the drug.

The choice of drug administration methods should be tailored to individual needs, and further research evidence is essential.

### 4.6 Clinical translational significance

A nomogram for hyponatremia risk assessment was constructed based on the two-predictor multivariate model (Figure 6). This nomogram is intended solely as a hypothesis-generating tool and a visual summary of the regression model. It was derived from a single-center cohort of 90 patients and has not undergone external validation. Accordingly, it should not be used for clinical decision-making. Future multicenter studies with larger sample sizes are required to validate its predictive accuracy before any consideration of clinical application.

### 4.7 Innovations and limitations

This study is one of the few real-world investigations specifically examining terlipressin safety in an exclusively elderly (≥60 years) EGVB cohort, building upon previous work in this area. Additionally, it provides an exploratory comparison of safety profiles between intermittent intravenous bolus injection and continuous pump infusion in elderly patients, offering preliminary data for future prospective studies.

This study has several limitations. First, the study did not meet its pre-specified sample size (enrolled 90 vs. target 96). Although a *post hoc* precision analysis confirmed that the primary estimate remains acceptably precise, the study is underpowered for secondary endpoints—including the risk factor analysis and the exploratory comparison of administration methods. Readers should interpret these findings with caution. Second, the single-center retrospective design introduces selection bias, as extremely critically ill patients might not be included due to early mortality, leading to “survivor bias,” and information bias, since missing urine output data for some patients could result in undiagnosed AKI. Third, and importantly, fluid management data—including the type, volume, and rate of fluid resuscitation, as well as diuretic use—were not recorded. Dilutional hyponatremia, the primary safety outcome of this study, is mechanistically driven by positive water balance. Therefore, the observed hyponatremia rate may partially or substantially reflect fluid management practices rather than terlipressin pharmacology alone. We cannot attribute the observed hyponatremia entirely to terlipressin. Fourth, the continuous infusion group has a relatively small sample size of 24 cases, limiting the statistical power of subgroup analyses. Consequently, the conclusions are exploratory suggestions. To address confounding, we conducted a sensitivity analysis using propensity score matching (PSM), and the results post-matching showed a similar numerical pattern. Fifth, the absence of a control group prevents direct comparison of the advantages and disadvantages of different regimens. Sixth, we did not collect long-term follow-up data, such as the 90-day readmission rate or long-term survival rate. Seventh, a pharmacoeconomic evaluation was not performed. Eighth, the small number of AKI events allowed only for descriptive reporting. Furthermore, AKI diagnosis relied exclusively on serum creatinine changes, as urine output data were inconsistently recorded. This constitutes a systematic bias toward underestimating the true incidence of AKI, particularly less severe Stage 1 cases that may present with oliguria before a rise in creatinine. Ninth, the potential for pharmacokinetic or pharmacodynamic interactions between terlipressin and omeprazole was not evaluated. Omeprazole is a moderate inhibitor of CYP2C19 and CYP3A4, and while no major interaction with terlipressin has been clinically established, a subtle effect on drug metabolism in patients with advanced liver disease cannot be entirely excluded. This warrants attention in future studies. Future research should involve multicenter prospective studies with larger sample sizes, incorporate long-term follow-up data, include detailed fluid management records, and assess potential drug-drug interactions to validate the conclusions of this study.

## 5 Conclusion

Terlipressin demonstrates a clear hemostatic effect in elderly patients with liver cirrhosis and esophagogastric variceal bleeding (EGVB) when administered with standard background therapy. However, it is associated with a relatively high incidence of hyponatremia. Patients classified as Child-Pugh class C or those with low baseline serum sodium levels require enhanced monitoring of serum sodium. The impact of administration method on hyponatremia requires further investigation.

## Data Availability

The original contributions presented in the study are included in the article/supplementary material, further inquiries can be directed to the corresponding author.

## References

[B1] Arkle T. Lam S. Bowers D. (2020). Hyponatraemia during terlipressin therapy: a retrospective cohort study. Frontline Gastroenterol. 12 (2), 108–113. 10.1136/flgastro-2020-101440 33613941

[B2] Arora V. Choudhary S. P. Maiwall R. Vijayaraghavan R. Jindal A. Kumar G. (2023). Low-dose continuous terlipressin infusion is effective and safer than intravenous bolus injections in reducing portal pressure and control of acute variceal bleeding. Hepatol. Int. 17 (1), 131–138. 10.1007/s12072-022-10416-6 36542261

[B3] Belcher J. M. Parada X. V. Simonetto D. A. Juncos L. A. Karakala N. Wadei H. M. (2022). Terlipressin and the treatment of hepatorenal syndrome: how the CONFIRM trial moves the story forward. Am. J. Kidney Dis. 79 (5), 737–745. 10.1053/j.ajkd.2021.08.016 34606933

[B4] Biggins S. W. Angeli P. Garcia-Tsao G. Ginès P. Ling S. C. Nadim M. K. (2025). Erratum: diagnosis, evaluation, and management of ascites, spontaneous bacterial peritonitis and hepatorenal syndrome: 2021 practice guidance by the American association for the study of liver diseases. Hepatology 82 (6), E160. 10.1097/HEP.0000000000000920 40966419

[B5] Corsonello A. Pedone C. Incalzi R. A. (2020). Age-related pharmacokinetic and pharmacodynamic changes and related risk of adverse drug reactions. Curr. Med. Chem. 17 (6), 571–584. 10.2174/092986710790416326 20015034

[B6] de Franchis R. Bosch J. Garcia-Tsao G. Reiberger T. Ripoll C. Baveno VII Faculty (2022). Baveno VII – renewing consensus in portal hypertension. J. Hepatol. 76 (4), 959–974. 10.1016/j.jhep.2021.12.022 35120736 PMC11090185

[B7] Elsebaey M. A. Elashry H. Elbedewy T. A. Elhadidy A. A. Esheba N. E. Ezat S. (2018). Predictors of in-hospital mortality in a cohort of elderly Egyptian patients with acute upper gastrointestinal bleeding. Med. Baltim. 97 (16), e0403. 10.1097/MD.0000000000010403 29668596 PMC5916675

[B8] European Association for the Study of the Liver (2022). EASL clinical practice guidelines on prevention and management of bleeding in cirrhosis. J. Hepatol. 76 (5), 1150–1184. 10.1016/j.jhep.2021.12.003 35300861

[B9] Garcia-Tsao G. Abraldes J. G. Rich N. E. Wong V. W. S. (2024). AGA clinical practice update on the use of vasoactive drugs and intravenous albumin in cirrhosis: expert review. Gastroenterology 166 (1), 202–210. 10.1053/j.gastro.2023.10.013 37978969

[B10] Glassock R. J. Rule A. D. (2022). Aging and the kidney: anatomy, physiology, and consequences for the development and progression of chronic kidney disease. Nephron 146 (3), 193–200. 10.1159/000519779

[B26] Han X. Li J. Yang J. M. Gao M. Wang L. (2019). A retrospective analysis of hyponatremia during terlipressin treatment in patients with esophageal or gastric variceal bleeding due to portal hypertension. JGH Open 4 (3), 368–370. 10.1002/jgh3.12254 32514438 PMC7273692

[B27] Hassan M. Merza N. Nawras Y. Bahbah E. I. Al-Hillan A. Ahmed Z. (2023). Continuous vs. intermittent terlipressin infusion for portal hypertension: a systematic review and meta-analysis. Ann. Med. Surg. (Lond) 85 (10), 5001–5010. 10.1097/MS9.0000000000001261 37811089 PMC10553058

[B11] Jindal A. Singh H. Kumar G. Arora V. Sharma M. K. Maiwall R. (2024). Early versus standard initiation of terlipressin for acute kidney injury in ACLF: a randomized controlled trial (eTerli Study). Dig. Dis. Sci. 69 (6), 2204–2214. 10.1007/s10620-024-08423-8 38637454

[B12] Kaplan D. E. Ripoll C. Thiele M. Fortune B. E. Simonetto D. A. Garcia-Tsao G. (2024). AASLD practice guidance on risk stratification and management of portal hypertension and varices in cirrhosis. Hepatology 79 (5), 1180–1211. 10.1097/HEP.0000000000000746 37870298

[B13] Kumar R. Kumar S. Prakash S. (2023). Acute variceal bleeding: a review of current management and outcomes. World J. Hepatol. 15 (7), 867–885. 10.4254/wjh.v15.i7.867 37547030 PMC10401411

[B16] Liu D. Testro A. Majumdar A. Sinclair M. (2025). The current applications and future directions of terlipressin. Hepatol. Commun. 9 (4), e0685. 10.1097/HC9.0000000000000685 40178480 PMC11970894

[B17] Maher D. Ailabouni N. Mangoni A. A. Wiese M. D. Reeve E. (2021). Alterations in drug disposition in older adults: a focus on geriatric syndromes. Expert Opin. Drug Metab. Toxicol. 17 (1), 41–52. 10.1080/17425255.2021.1839413 33078628

[B18] Rehman H. Rehman S. T. Zulfiqar S. Awan S. Abid S. (2024). Real-world comparison of terlipressin vs. octreotide as an adjuvant treatment in the management of variceal bleeding. Sci. Rep. 14 (1), 6692. 10.1038/s41598-024-56873-x 38509184 PMC10954665

[B19] Şenkaya A. Çelik F. Kurtulmuş Akgün İ. Aslanov S. Uysal A. Buyruk A. M. (2025). Terlipressin treatment for acute esophageal variceal bleeding: bolus or infusion? Turk. J. Gastroenterol. 37 (2), 208–214. 10.5152/tjg.2025.25265 41669923 PMC12910312

[B20] Thomson M. J. Tapper E. B. Lok A. S. F. (2022). Managing the older patient with cirrhosis. Hepatology 75 (3), 748–766. 10.1002/hep.32229

[B22] Velez J. C. Q. Wong F. Reddy K. R. Sanyal A. J. Vargas H. E. Curry M. P. (2023). The effect of terlipressin on renal replacement therapy in patients with hepatorenal syndrome. Kidney 4 (8), 1030–1038. 10.34067/KID.0000000000000161 37143199 PMC10482068

[B23] Wong F. Pappas S. C. Curry M. P. Reddy K. R. Rubin R. A. Porayko M. K. (2021). Terlipressin plus albumin for the treatment of type 1 hepatorenal syndrome. N. Engl. J. Med. 384 (9), 818–828. 10.1056/NEJMoa2008290 33657294

[B24] Xiao J. Wang F. Wong N. K. He J. Zhang R. Sun R. (2019). Global liver disease burdens and research trends: analysis from a Chinese perspective. J. Hepatol. 71 (1), 212–221. 10.1016/j.jhep.2019.03.004 30871980

[B25] Zanetto A. Shalaby S. Feltracco P. Gambato M. Germani G. Russo F. P. (2021). Recent advances in the management of acute variceal hemorrhage. J. Clin. Med. 10 (17), 3818. 10.3390/jcm10173818 34501265 PMC8432221

